# Salinity Stress Mechanisms in *Sepia esculenta* Larvae Revealed by Integrated Biochemical and Transcriptome Analyses

**DOI:** 10.3390/biology14101338

**Published:** 2025-09-30

**Authors:** Yancheng Zhao, Xueyu Zhu, Jingzhao Zhang, Weijun Wang, Cuiju Cui, Xin Tan, Xiumei Liu, Xiaohui Xu, Zan Li, Jianmin Yang

**Affiliations:** 1 School of Fisheries, Ludong University, Yantai 264025, China; zycheng6633@163.com (Y.Z.); 15735439008@163.com (X.Z.); 18863872307@163.com (J.Z.); wwj2530616@163.com (W.W.); cuicuiju@163.com (C.C.); ladderup@126.com (J.Y.); 2 College of Life Sciences, Yantai University, Yantai 264005, China; Tanx213814@163.com (X.T.); xiumei0210@163.com (X.L.); 3 College of Fisheries and Life Science, Shanghai Ocean University, Shanghai 201306, China

**Keywords:** *Sepia esculenta*, salinity stress, biochemical quantitative experimental analysis, transcriptome, gene set enrichment analysis

## Abstract

**Simple Summary:**

Our study showed that chemokine signaling pathways, MAPK signaling pathways, and cell cycle were identified in *S. esculenta* under different salinity stress. In particular, we identified the core genes, including *NFKBIA*. These findings suggest that *NFKBIA*, the core gene of this gene set, exhibits a central regulatory role, which potentially contributes to alleviating oxidative stress and regulating immune function. This study provides valuable insights for aquaculture practices guided by immunological principles.

**Abstract:**

The stable marine environment is conducive to the development of the aquaculture industry. However, with the change of seawater salinity in recent years, it has had a great impact on the survival and breeding of cephalopods such as *Sepia esculenta*. In this study, biochemical measurement and transcriptome sequencing were performed on the larvae of *S. esculenta* after different salinity stresses (salinity of 20 ppt and 40 ppt), and the reliability of transcriptome results was proved by physiological indexes. We performed Gene Ontology (GO), Kyoto Encyclopedia of Genes and Genomes (KEGG) enrichment analysis, and gene set enrichment analysis (GSEA) on all annotated genes, and gene sets were identified, including chemokine signaling pathways, MAPK signaling pathways, and cell cycle pathways. Finally, we constructed the protein-protein interaction networks (PPI) between the core genes in these gene sets and differentially expressed genes (DEGs) to identify key genes, including *NFKBIA*. Among them, the *NFKBIA* is not only a core gene in the chemokine signaling pathway gene set under four stresses but also has a high number of protein interactions. We speculate that this gene may have important immunomodulatory functions in the face of different time and salinity stresses. The results of our study explored the molecular mechanism of *S. esculenta* in the face of environmental stress, revealed the key molecular regulatory pathways for its survival and adaptation under complex environmental pressures, and may provide insights relevant to the development of *S. esculenta* pond culture.

## 1. Introduction

For aquaculture, a stable and suitable marine environment is required as support [1,2]. However, in recent years, with the change of environment, the change of seawater index is accompanied by, such as, the fluctuation of seawater salinity in different sea areas [3,4]. Changes in seawater salinity caused by environmental changes pose a great threat to some marine organisms [5,6]. For example, increased salinity can trigger severe stress responses in *marine organisms*, sometimes leading to mortality [7]. Marine organisms are very susceptible to salinity changes, as even minor fluctuations can disrupt osmoregulatory mechanisms in aquatic organisms, ultimately compromising their viability and reproduction through osmotic stress [8]. Changes in ocean salinity could also have a significant influence on the vital activities of marine mollusks [9]. In some of the earlier studies, Knowles et al. [10] investigated physiological changes in Crassostrea gigas under low salinity stress. Their research results revealed that when stimulated by low salt concentration, the cells of *Crassostrea gigas* were negatively affected and obvious tissue lesions appeared [10]. Li et al. demonstrated that the increase in salinity stress enhanced the apoptosis of *S. esculenta* to reduce the tissue damage caused by the increase in seawater salinity [11].

High-throughput sequencing technology has advanced quickly in past years. Using this technology can make the analysis of organisms more efficient and accurate [12,13]. And in previous studies, this technique has been used to analyze the biological processes of aquatic organisms. For example, Zhao et al. found that the dual stress of high temperature and copper can cause DNA damage in *S. esculenta*, and there will be a serious inflammatory response by transcriptome analysis [14]. Through transcriptome analysis, Liu et al. found that *Crassostrea gigas* participates in the immune function of the body by regulating the MAPK signaling pathway and PIK3-Akt signaling pathway in the face of *Vibrio alginolyticus* invasion [15]. Building on these studies, the present work explores the biological responses of *S. esculenta* to different salinity stresses using transcriptome sequencing.

Cephalopods are an important part of marine ecosystems and have important economic value for fisheries [16]. Among them, *S. esculenta* is a kind of squid with high economic and commercial value, mainly distributed in the East China Sea, South China Sea, Yellow Sea, and Bohai Sea coastal waters [17]. Large environmental fluctuations significantly increase mortality in *S. esculenta*, leading to substantial aquaculture losses, which will cause huge losses to the aquaculture industry. Therefore, this study selected *S. esculenta* larvae for experiments to explore the influences of different time and salinity stress on mollusks and enriched the biological theory of *S. esculenta* in the face of adversity.

In the research, we first analyzed the influences of different time and salinity stresses on the activities of oxidative stress-related enzymes and immune-related enzymes in *S. esculenta*. The biological processes of *S. esculenta* larvae under different salt stresses were explored by sequencing. The larvae of *S. esculenta* under different salinity stress were sampled, and RNA sequencing was performed, and the quality was controlled. Then, the identified DEGs were used for GO and KEGG enrichment analysis. The core genes in the significantly enriched gene set were combined with the DEGs in the corresponding KEGG pathway to construct the PPI. After that, we took the protein interaction number as the main reference factor to explore the key genes of *S. esculenta*, such as NFKBIA. Previous studies have shown that NFKBIA can reduce the stimulation of external stress on the species itself to a certain extent and can respond to stress through its own protective mechanism [18,19]. Finally, we used qRT-PCR to verify the quality of the sequencing results. In view of current environmental changes and the prospects for pond culture of *S. esculenta*, this study provides insights into the effects of salinity stress on larvae, offering a theoretical basis for large-scale aquaculture.

## 2. Materials and Methods

### 2.1. Acquisition and Processing of Experimental Samples

In the research, we collected adult *S. esculenta* (weight: 348.93 ± 11.23 g, mantle length: 13.79 ± 0.20 mm) from the sea area near Qingdao in mid-July. After short-distance transportation, it was temporarily cultured in a culture pond to adapt to the environment. The seawater temperature in the pond was 21 ± 1 °C, and the salinity was 30.6 ± 1 °C. Subsequently, eggs were harvested from the pool every day using an attachment net and placed in a perforated plastic basin. After the eggs were collected, the culture basin was transferred to a designated acclimation pond implementing continuous flow-through seawater exchange with supplemental aeration, maintaining physicochemical parameters (temperature: 21 ± 1 °C; salinity: 30 ± 0.5 ppt; pH: 8.1 ± 0.1; dissolved oxygen: 5.7 ± 0.2 mg/L) equivalent to maternal pond conditions. We injected a total of 100 L of seawater into six square culture barrels with a capacity of 120 L. The two groups were set as the low salinity stress group (salinity of 20 ppt) and high salinity stress group (salinity of 40 ppt), and the other groups did not make any changes. Approximately 100 *S. esculenta* larvae in each group were placed and sampled at 4 h and 24 h. Seven experimental groups were set up, which were the normal growth control group (C_0h, C_4h, and C_24h), the low salinity stress group (SAL20_4h and SAL20_24h), and the high salinity stress group (SAL40_4h and SAL40_24h). Nine larvae were randomly selected at each time point in each experimental group, and three random samples were mixed together to generate three biological replicates. All samples were placed in test tubes and immediately frozen in liquid nitrogen.

### 2.2. Determination of Enzyme Activity and Related Product Content

The activity of Acid Phosphatase (ACP), Alkaline Phosphatase (AKP), Glutathione S-Transferase (GST), Superoxide Dismutase (SOD), and the content of Malondialdehyde (MDA) were measured by corresponding kits (Nanjing Jiancheng Bioengineering Institute, Nanjing, China). All experimental determinations were performed according to the kit instructions, and each measurement was repeated three times to ensure accuracy.

### 2.3. Transcriptome Data Processing and Analysis

The TRIzol method was used to extract total RNA, followed by library preparation. Paired-end sequencing was performed on the Illumina TruSeq HiSeq 4000 platform (New England Biolabs, Ipswich, MA, USA). Raw reads underwent rigorous quality filtering: adapter-containing sequences were removed, reads exceeding 10% undetermined bases (N ratio) were discarded, and low-quality reads were eliminated (defined as those with >50% bases showing Qphred ≤ 20). The resulting clean reads were subjected to quality assessment through FastQC (v0.12.0), with Q20 and Q30 average scores calculated as key quality metrics. Reference genome alignment was executed using HISAT2 (v2.2.1) with default parameters. Differential expression analysis was performed using DESeq2 (v1.38.3) with a negative binomial distribution model. The analytical workflow included data import and construction of the DESeqDataSet (dds) object; Dispersion estimation through DESeq function execution; and Comparative expression analysis between experimental groups (low salinity vs. control and high salinity vs. control). DEGs were identified using a significance threshold of |Log2 Fold Change| ≥ 1 and *p*-Value ≤ 0.05 after multiple testing correction.

### 2.4. Analysis of DEGs

In the study, the expression of DEGs was displayed by using volcano plots, heatmaps, and Venn diagrams. The volcano plots show the distribution of gene expression. The points with significant differences are given different colors by using the multiplicity of differences between groups to show the significance of the differences. The log10 of fold change values was used on the x-axis, and the −log10 of *p*-values was plotted on the y-axis. The clustering heatmap shows the expression patterns of DEGs in different samples. Z-score was used to standardize the FPKM data of DEGs and selected for horizontal clustering. The number of DEGs at different time points was shown by a Venn diagram.

### 2.5. Functional Analysis of DEGs

The DAVID database (https://david.ncifcrf.gov/tools.jsp, accessed on 7 November 2024) was used to perform GO and KEGG enrichment analysis on DEGs, and DEGs were annotated into GO terms and KEGG signaling pathways. Then, all the annotated genes in SAL20_4h, SAL20_24h, SAL40_4h, and SAL40_24h were used for GSEA analysis (R version 4.2.2), and the genes in the GO function enrichment term and the signal pathway of KEGG enrichment analysis were used as gene sets for analysis. 

### 2.6. Protein-Protein Interaction Networks Analysis

Build the PPI networks by using the STRING v11.5 online website. The core genes in the pathway gene set with higher enrichment scores in GSEA-KEGG analysis and DEGs in KEGG enrichment analysis were combined to construct the protein-protein interaction networks. A minimum interaction score of 0.15 was applied. According to the number of protein interactions involved in the gene as the main reference factor, the key genes after high and low salt stress at different time points were analyzed.

### 2.7. qRT-PCR Validation

The accuracy of the sequencing results of this study was verified by qRT-PCR. Our qRT-PCR validation selected DEGs with more protein interaction numbers in the gene sets. The verified DEGs and their sequences are shown in Table 1. According to the manufacturer’s instructions, the remaining RNA from the library was synthesized into cDNA using reverse transcriptase (Promega, Madison, WI, USA) (42 °C, 1 h; 70 °C, 10 min). The qPCR program and final reaction volume were determined according to the instructions for the use of ABI 7500 (Thermo Fisher Scientific, Waltham, MA, USA) with SYBR^®^ Premix Ex Taq™ (Takara, Dalian, China), and β-actin was used as an internal reference. The expression of target genes was calculated using the 2^−△△CT^ comparative Ct value method. Differential expression of target genes was expressed as a fold change relative to the internal reference gene.

### 2.8. Statistical Analysis

All experiments in this study were repeated at least three times. To ensure maximum randomization during sample processing, nine larval specimens were randomly pooled from a cohort of 100 individuals spanning various experimental groups. This strategic mixing approach enhances the representativeness of biological samples while maintaining experimental rigor. And when evaluating the differences between groups, *p* < 0.05 was considered statistically significant.

## 3. Results

### 3.1. Sequencing Results

The sequencing results of different samples of *S. esculenta* were analyzed. The average scores of both Q20 and Q30 were above 90%. This indicates that the sequencing results can be used for subsequent processing (Table 2).

### 3.2. Enzyme Activity and Peroxide Content

**Figure 1:**
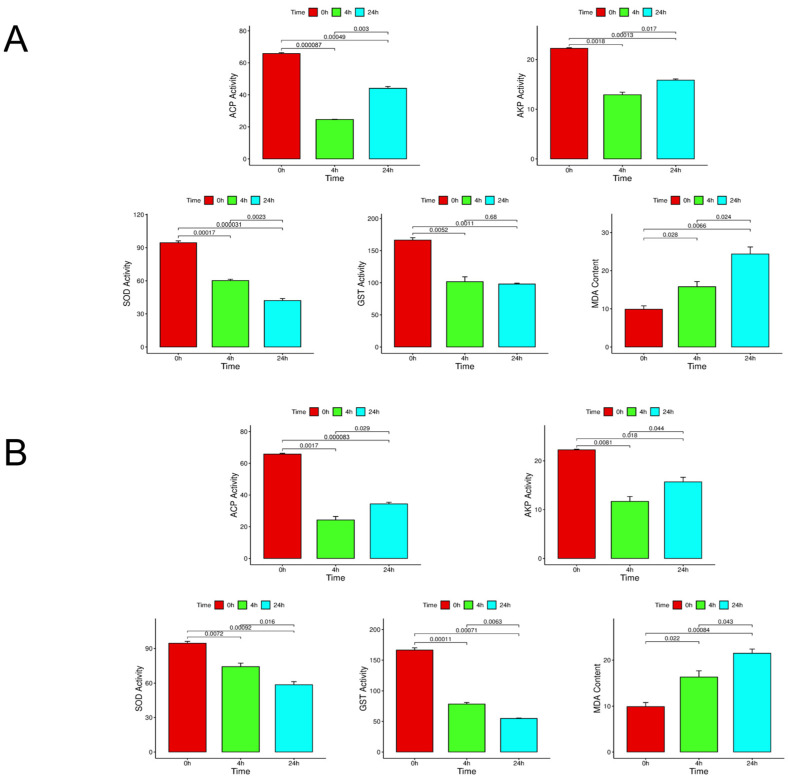
The diagram shows the changes in enzyme activity and peroxide content under different stresses. (**A**): ACP, AKP, SOD, GST activity, and MDA content at different times under low salt stress. (**B**) Enzyme activity and peroxide content under high salt stress. Ensure three biological replicates, and *p* < 0.05 as a sign of significant difference.

The results of enzyme activity experiments showed that the activities of ACP and AKP, two immune-related enzymes, decreased significantly in the face of high salinity and low salinity stress. With temporal progression, compared with 4 h, the activity of the two enzymes even showed an upward trend at 24 h. The activities of GST and SOD, two oxidative stress-related enzymes, showed significant differences at 4 h and 24 h compared with 0 h in the face of three different stresses. MDA as a product of peroxidation in the face of stress, with the extension of time, has a significant upward trend. Figure 1 shows the changes in enzyme activity and peroxide content under different stresses.

### 3.3. Identification of DEGs

**Figure 2:**
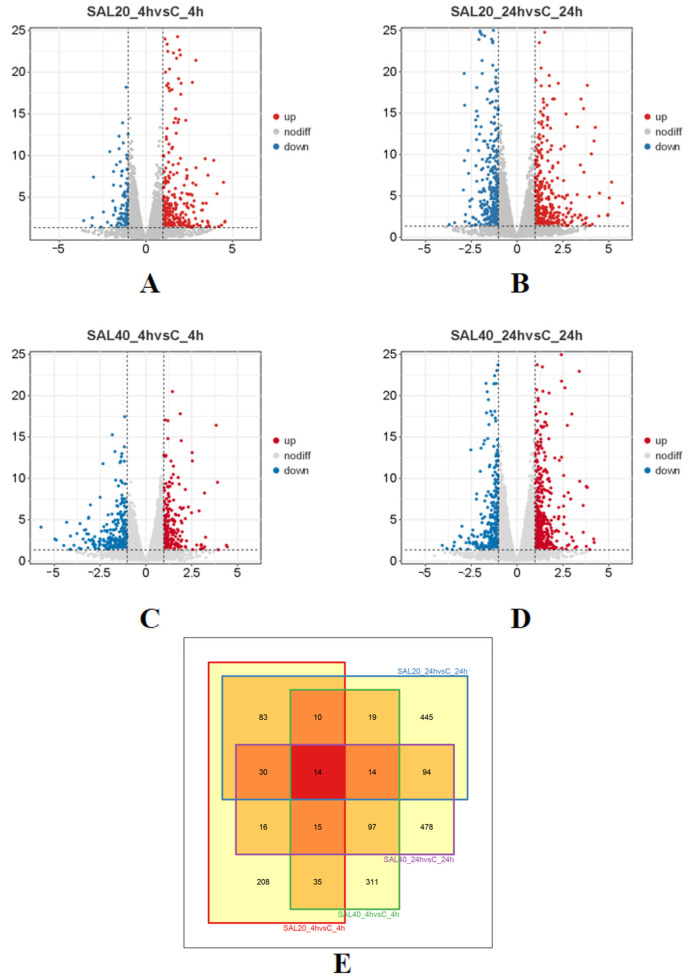
Volcano diagram and Venn diagram of DEGs (|Log2FC| ≥ 1, *p* ≤ 0.05). (**A**) Gene expression between SAL20_4h samples. The up-regulated genes were expressed in orange, and the down-regulated genes were expressed in blue. Each point represents a gene. (**B**) Gene expression between SAL20_24h samples. (**C**) Gene expression between SAL40_4h samples. (**D**) Gene expression between SAL40_24h samples. (**E**) The number of DEGs in SAL20_4h, SAL20_24h, SAL40_4h, and SAL40_24h samples. Red indicates the number of co-expressed DEGs.

By comparing the samples of SAL20_4h, SAL20_24h, SAL40_4h, and SAL40_24h with the control group, 411, 709, 515, and 758 DEGs were identified with |log2 (fold change)| ≥ 1 and *p*-value ≤ 0.05 as thresholds (Figure 2). Subsequently, the DEG was analyzed by a Venn diagram (Figure 2E). The intersection of the Venn diagram produced 14 key genes, including *SMP_049250*, *LOC114955324*, *KLF5*, *COL12A1*, *PML*, *SLIT2*, *CYP10*, *FOS*, *FMRFAR*, *FBXO4*, *NCL-1*, *COL14A1*, *APLNRB*, and *NOS1*. It is worth noting that these 14 genes concurrently showed differential expression in four experimental groups, suggesting their potential critical functional roles in responding to temporal variations and varying salinity conditions.

### 3.4. Clustering Heatmap Analysis

**Figure 3:**
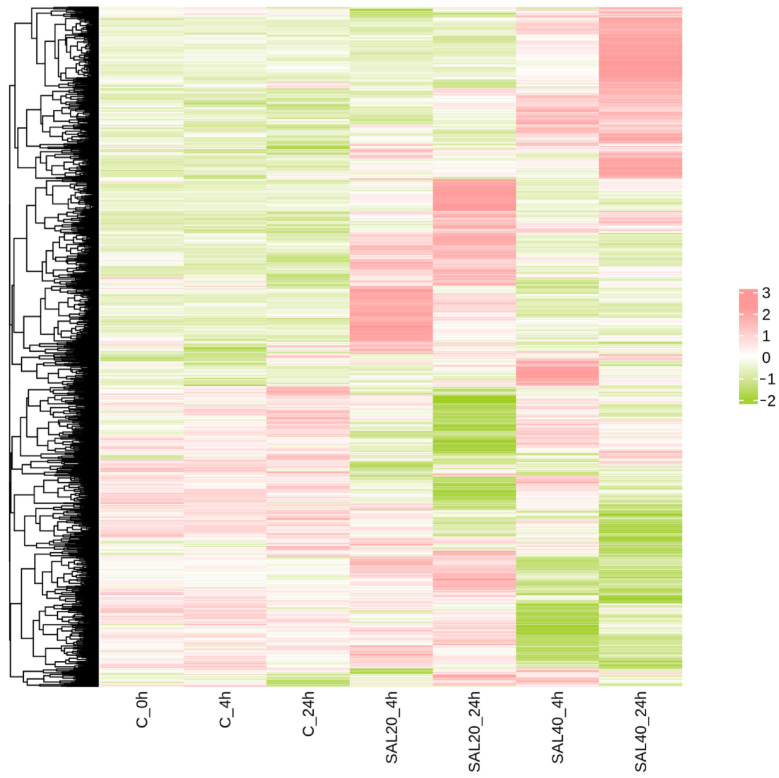
Clustering heat map of DEGs. DEGs were differentially expressed in different experimental groups. Green to red indicates the relative expression from high to low. Color represents the result of Z-score processing of the FPKM values of all DEGs.

The intersection of DEGs at different times and different salinity stresses was analyzed by cluster heatmap (Figure 3). The findings indicated that there were basically the same expression patterns between the three groups of blank control groups at different times. Compared with the 4 h blank group, the 4 h experimental groups under different salinity stress showed significant differences. There was also a huge difference between the 24 h experimental group and the control group.

### 3.5. Functional Analysis of DEGs

**Figure 4:**
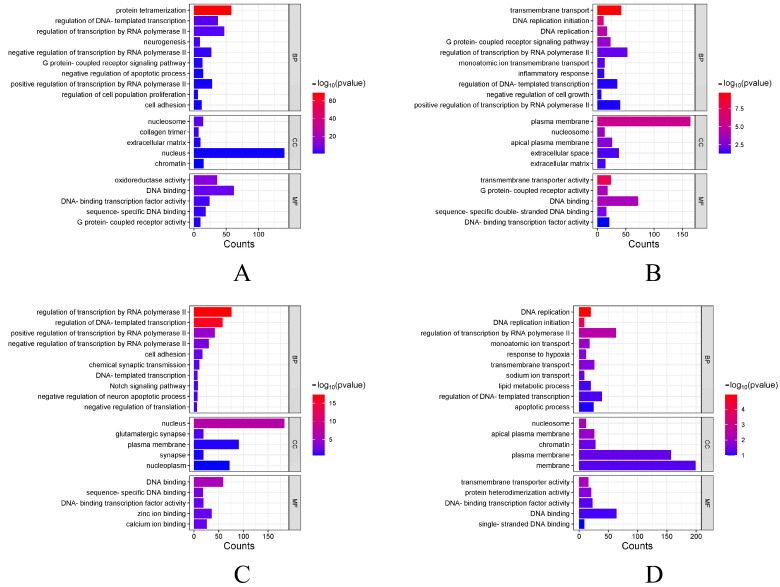
GO enrichment analysis of DEGs. (**A**) GO term enriched under SAL20_4h stress. The vertical axis represents different terms, and the horizontal axis represents the number of genes. (**B**) GO term enriched under SAL20_24h stress. (**C**) GO term enriched under SAL40_4h stress. (**D**) GO term enriched under SAL40_24h stress. With a *p*-value < 0.05 as the selection criteria, and in line with the species we studied.

**Figure 5:**
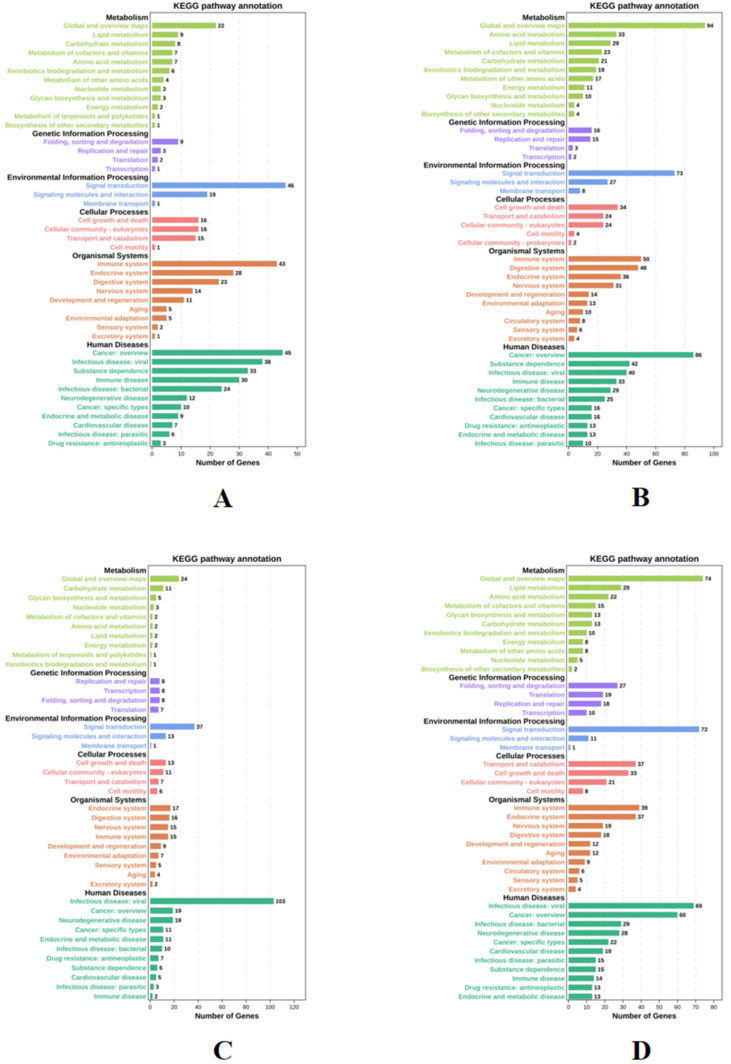
Level-2 KEGG signaling pathways of DEGs. (**A**) KEGG signaling pathways were enriched with DEGs under SAL20_4h stress. (**B**) KEGG signaling pathways were enriched with DEGs under SAL20_24h stress. (**C**) KEGG signaling pathways are enriched using DEGs under SAL40_4h stress. (**D**) KEGG signaling pathways are enriched using DEGs under SAL40_24h stress. With a *p*-value < 0.05 as the selection criteria, and in line with the species we studied.

The functions of DEGs under four different stress conditions were explored by GO enrichment analysis, and DEGs are enriched into three categories. Figure 4 shows the top 10 biological processes and the top 5 cellular components and molecular functions. Among them, a large number of terms are associated with DNA replication, binding, and regulation of RNA polymerase II transcription. After that, KEGG pathway enrichment was analyzed for DEGs across four experimental groups. Figure 5 shows the enrichment of DEGs in the Level-2 KEGG signaling pathway. Among them, in the signal transduction and immune system, a large number of DEGs were enriched in the four experimental groups. The enrichment of DEGs in the level-3 KEGG pathway under different time and salinity stresses was shown in the annex table (Supplementary Table S1).

### 3.6. GSEA Analysis

**Figure 6:**
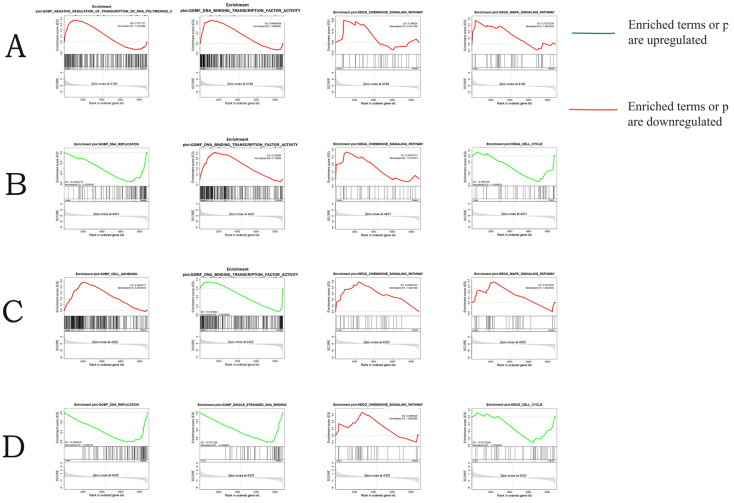
GSEA analysis results. (**A**) Significantly enriched gene sets under SAL20_4h stress. (**B**) Significantly enriched gene sets under SAL20_24h stress. (**C**) Gene sets significantly enriched under SAL40_4h stress. (**D**) Gene sets significantly enriched under SAL40_24h stress.

GSEA-GO and GSEA-KEGG analyses were performed on all annotated genes in four different experimental groups. This comprehensive analysis enables us to determine the most significantly enriched gene sets at different time points and under different salinity stress conditions, providing valuable information for our in-depth understanding. Figure 6 shows that negative regulation of transcription by RNA polymerase II, sequence-specific DNA binding, and the MAPK signaling pathway are significantly enriched in SAL20_4h. DNA replication, DNA-binding transcription factor activity, chemokine signaling pathway, and cell cycle are significantly enriched at SAL20_24h. Cell adhesion, DNA-binding transcription factor activity, the chemokine signaling pathway, and the MAPK signaling pathway are significantly enriched in SAL40_4h. Finally, DNA replication, single-stranded DNA binding, the chemokine signaling pathway, and cell cycle are significantly enriched in SAL40_24h. The MAPK signaling pathway was significantly enriched in SAL20_4h and SAL40_4h, and the cell cycle was significantly enriched in SAL20_24h and SAL40_24h. More importantly, the chemokine signaling pathway is significantly enriched in SAL20_24h, SAL40_4h, and SAL20_24h. Although there is no significant enrichment in SAL20_4h, its core genes still contribute greatly to its gene set, so it has great biological significance. Therefore, the core genes of the chemokine signaling pathway in the GSEA analysis of the four experimental groups are compared, and it is found that *NFKBIA* appeared as a core gene in the four experimental groups. Therefore, we speculate that *NFKBIA* plays a more critical role under different time and salinity stress.

### 3.7. Analysis of PPI

**Figure 7:**
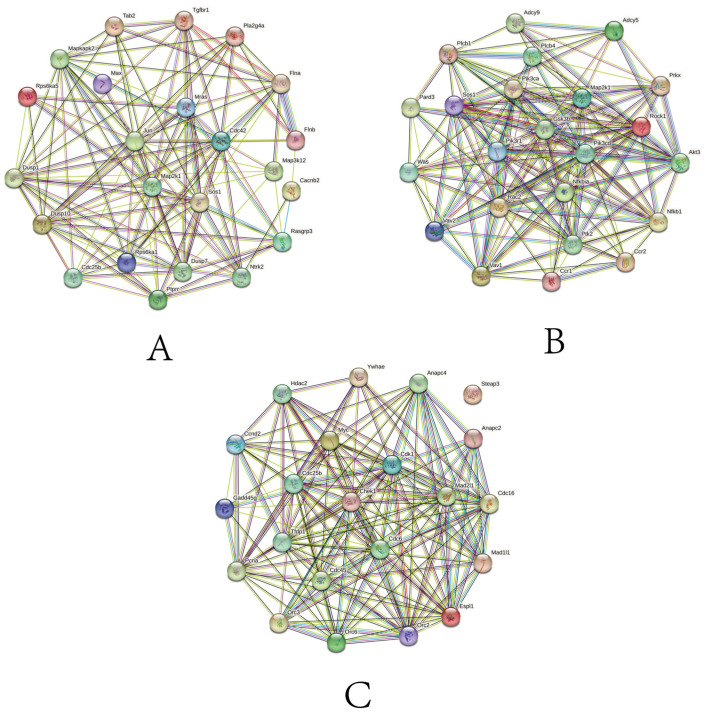
PPI network of DEGs (a minimum interaction score of 0.15 was applied). Genes are represented by circles. (**A**) PPI network composed of core genes in chemokine signaling pathway gene set. (**B**) PPI network composed of core genes and DEGs in MAPK signaling pathway gene set. (**C**) PPI network composed of core genes and DEGs in cell cycle gene set.

In the chemokine signaling pathway, *GSK3B*, *MAP2K1*, and PI3K family genes, such as *PIK3CD*, *PIK3CA*, and *PIK3R1*, have higher protein interactions. More importantly, *NFKBIA* is the core gene in the gene set and also has a high number of protein interactions. Therefore, we speculate that this gene may have a more important function in the face of different time and salinity stresses. In the MAPK signaling pathway, *JUN*, *MAP2K1*, and *DUSP* family genes, such as *DUSP1*, *DUSP10*, and *DUSP7*, have higher protein interaction numbers. In the cell cycle signaling pathway, *CDK1*, *CHEK1*, and CDC family genes, such as *CDC6*, *CDC16*, *CDC25B*, and *CDC45*, have higher protein interactions. Among them, *MAP2K1* appears as a core gene in both pathways and may also play a key function in the face of salinity stress (Figure 7).

### 3.8. qRT-PCR Validation of DEGs

**Figure 8:**
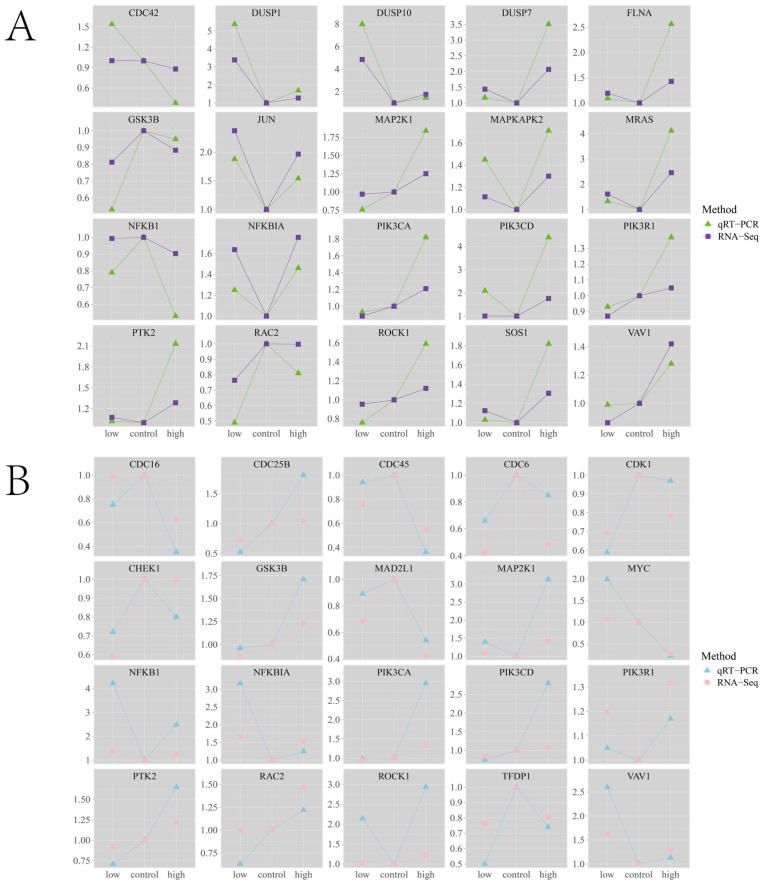
qRT-PCR validation of core gene. (**A**) The core gene with the highest number of protein interactions in chemokine signaling pathway gene set and MAPK signaling pathway gene set. The abscissa represents the way of stress, and the ordinate represents the Fold change of 4h stress. (**B**) The core gene with the highest number of protein interactions in chemokine signaling pathway gene set and cell cycle gene set. The abscissa represents the way of stress, and the ordinate represents the Fold change of 24 h stress.

The quantitative verification results proved the accuracy of the sequencing results. The fold changes under different experimental conditions were consistent, indicating that the change trend of DEGs was consistent (Figure 8).

## 4. Discussion

### 4.1. Expression and Functional Enrichment Analysis of DEGs

In this research, four experimental groups of SAL20_4h, SAL20_24h, SAL40_4h, and SAL40_24h were set up to form a union of 1861 DEGs. And with the extension of time under different salinity stress, the number of DEGs also increased. The clustering heat map selects the filtered DEGs for drawing. Studies have shown that under different salinity stress, at 4 h and 24 h, a considerable number of DEGs have different expression levels. After that, 411, 709, 515, and 758 DEGs identified by SAL20_4h, SAL20_24h, SAL40_4h, and SAL40_24h were respectively subjected to GO and KEGG functional enrichment analysis. GO enrichment analysis showed that most of the terms were related to DNA replication, binding, and regulation of RNA polymerase II transcription. The significant enrichment of ‘ DNA replication ‘ as a basis for cell proliferation and genomic stability indicates that larvae enhance the replication process to reduce the harm caused by environmental stress under different salinity stimuli [20,21]. KEGG enrichment analysis showed that most DEGs were enriched in two Level-2 KEGG signaling pathways of signal transduction and the immune system in four different experimental groups. In the attached table, the three-level pathways of KEGG enrichment analysis are displayed. Among them, the MAPK signaling pathway is enriched in SAL20_4h, SAL20_24h, SAL40_4h, and SAL40_24h, and this pathway was important in the immune response of mollusks [22,23]. And in the three experimental groups, apoptosis was enriched, which means that the organism has immune-related problems [24]. In the SAL20_24h experimental group, this pathway was not enriched. It may be that the *S. esculenta* adapts to the stress of low salinity through its own regulation. The enrichment analysis of GO and KEGG shows that both high and low salinity seawater are likely to affect the growth and development of *S. esculenta* larvae. These results can provide a reference for the artificial breeding of *S. esculenta* to cope with different environmental changes.

### 4.2. Discussion on Enzyme Activity and Peroxide Content

Due to the lack of specific immune cells and related antibodies in mollusks, their humoral immunity primarily relies on non-specific enzymes or factors in the serum [25,26]. ACP enzyme and AKP enzyme are related to immunity and have important functions in immune response [27]. GST and SOD are important antioxidant enzymes that protect the body from oxidative stress [28,29]. MDA is an important product of peroxidation and is a key indicator of the level of oxidative stress in the body [30]. ACP and AKP were immune-related enzymes, and their enzyme activities showed a significant downward trend at 4 h. Therefore, we speculate that it may be because the body‘s immune system is difficult to adapt to this large-scale stress response in a short time. With the prolongation of time, the activities of ACP and AKP increased significantly at 24 h, indicating that with the prolongation of time, the body may regulate the expression of immune-related genes and other ways to make the body adapt to these three different stresses. GST and SOD as antioxidant enzymes, activity compared with 0 h, in 4 h and 24 h showed significant differences; the enzyme activity with the increase in time has been showing a significant downward trend, indicating that the three kinds of stress will lead to the body of *S. esculenta* antioxidant enzyme inhibition, thus weakening the *S. esculenta* larvae’s resistance to stress ability, resulting in *S. esculenta* antioxidant capacity being inhibited and significantly decreased. The content of MDA can measure the degree of oxidative damage to the body, so its accumulation is positively correlated with the level of oxidative stress. In our study, under three different stresses, the content of MDA showed a significant upward trend with temporal progression and reached the maximum at 24 h. It can be speculated that these stresses caused serious oxidative damage to the body of *S. esculenta.* The antioxidant defense system has also been damaged, and it is likely to have aggravated cell damage. These findings collectively highlight the critical role of immunity and oxidative balance in the stress response of mollusks, underscoring the intimate crosstalk between redox homeostasis and immune defense.

### 4.3. Chemokine Signaling Pathway

In the subsequent GSEA-GO analysis of all annotated genes, a large number of GO terms related to DNA replication, binding, and regulation of RNA polymerase II transcription were significantly enriched as gene sets under salinity stress at different times. In GSEA-KEGG analysis, the gene set of chemokine signaling pathway was significantly enriched in the three experimental groups of SAL20_24h, SAL40_4h, and SAL40_24h. Although not significantly enriched in SAL20_4h, the core genes of the gene set still have great biological significance. Therefore, in order to screen out more critical genes, PPI was constructed by constructing all the core genes in the GSEA analysis of the four experimental groups. Among them, *GSK3B*, *MAP2K1*, and PI3K family genes, such as *PIK3CD*, *PIK3CA*, and *PIK3R1*, have higher protein interaction numbers. Among them, the *PIK3CD* is involved in the immune response process and has an important function [31]. It is worth noting that *NFKBIA* also has a high number of protein interactions, and the chemokine signaling pathway in the four experimental groups is used as the core gene. *NFKBIA*-encoded IκBα protein is an important regulator of the NF-κB signaling pathway and has a significant function in coordinating various immune and inflammatory responses [32]. In previous studies, *NFKBIA* is directly involved in the regulation of the NF-κB signaling pathway and is a significant factor in maintaining the balance of NF-κB activity. NF-κB can enhance the antioxidant capacity of cells by regulating the expression of antioxidant genes [33,34]. In our experiment, by quantitatively analyzing the FPKM value of its expression level, it can be concluded that the gene showed an upward trend of expression under high and low salt stress at different times. This observed increase suggests a potential adaptive response where elevated *NFKBIA*/IκBα expression may contribute to fine-tuning NF-κB activity, potentially leading to enhanced cellular antioxidant defenses during salinity stress. Therefore, we speculate that the gene will increase its expression level in the face of salinity stress at different times so that the body‘s antioxidant defense ability can be improved, thereby helping the body to resist the damage caused by oxidation. In the GSEA-KEGG analysis of the four experimental groups, *NFKBIA* appeared as a core gene in the chemokine signaling pathway gene set; the consistent centrality of *NFKBIA* within the chemokine signaling pathway core gene sets across diverse salinity/time conditions underscores its likely significant role in *S. esculenta*’s molecular adaptation to osmotic challenge. Therefore, we infer that the upregulation of *NFKBIA* under salinity stress suggests its role in fine-tuning NF-κB activity to reinforce antioxidant defenses and modify immunophysiological adaptation in *S. esculenta*. The persistent enrichment of the chemokine signaling pathway underscores its fundamental role in orchestrating immune responses to different salinity stresses. Central genes such as *PIK3CD* and *NFKBIA* are pivotal in mediating inflammatory and antioxidant responses. Specifically, *NFKBIA*, through its regulation of NF-κB, modulates the expression of cytokines and antioxidants, thereby bridging innate immunity and oxidative stress management. This pathway is an important immunoregulatory pathway for immunophysiological adaptation of *S. esculenta*, facilitating survival under the condition of salinity fluctuation at different times through coordinated immune signaling and cellular defense mechanisms.

### 4.4. MAPK Signaling Pathway

The MAPK pathway regulates diverse disease-related processes, including oxidative stress, inflammatory response, cell proliferation, and apoptosis [35]. In the SAL20_4h and SAL40_4h experimental groups, the MAPK signaling pathway was significantly enriched as a gene set. In order to screen out more critical genes, we constructed a PPI network between all core genes in the GSEA analysis of SAL20_4h and SAL40_4h and all DEGs in the KEGG enrichment analysis. Among them, *JUN*, *MAP2K1*, and DUSP family genes, such as *DUSP1*, *DUSP10*, and *DUSP7*, have higher protein interaction numbers. Among them, *DUSP1* functions as a phosphatase responsible for dephosphorylating target enzymes, having the regulatory function in multiple immune signaling cascades [36,37]. *JUN* can reduce the degree of tissue oxidative damage by inhibiting the production of ROS [38]. As a pivotal regulatory gene in immune response mechanisms, *JUN* coordinates multiple signaling pathways within the immune regulatory network, demonstrating critical functions across diverse biological processes, including inflammatory regulation, cellular proliferation/differentiation of immune components, and transduction of immunological signals [39,40]. *MAP2K1* is a member of the bispecific protein kinase family. As a MAP kinase, it is expressed in different tissues. The protein kinase activates and regulates the enzyme activity of MAP kinase through extracellular and intracellular signaling pathways, thereby participating in a variety of physiological processes, including differentiation, proliferation, transcriptional regulation, and development [41]. The MEK1 protein encoded by *MAP2K1* has a significant function in the Ras/MAPK pathway that controls many cellular and developmental processes [42,43]. This induction pattern, coupled with redox homeostasis and immunomodulation, implicates these hubs in orchestrating rapid osmoregulatory adaptation through antioxidant defense and immune pathway fine-tuning. Importantly, *MAP2K1* appears as a core gene in both pathways and has a high number of protein interactions. We infer that the gene plays an important role in the regulation of cell-related processes in the face of salt stress, ensuring that the body can regulate cellular processes to help it adapt to the environment. In our research, the expression levels of the above genes were up-regulated in SAL20_4h and SAL40_4h. We speculated that the above genes may have an important function in the body‘s immunity and anti-oxidation in the face of short-term stress of different salinities. The enrichment of the MAPK pathway and the prominence of hub genes like *JUN*, *DUSP1*, and *MAP2K1* highlight a rapid immune-transcriptional reprogramming in response to acute salinity stress. These genes are integral to regulating inflammation, oxidative stress, and immune cell responses—processes essential for maintaining immunometabolic homeostasis.

### 4.5. Cell Cycle Pathway

In SAL20_24h and SAL40_24h, the gene set of the cell cycle was significantly enriched. In order to screen out more critical genes, the same analysis method as above was used. Among them, *CDK1*, *CHEK1*, and CDC family genes, such as *CDC6*, *CDC16*, *CDC25b*, and *CDC45*, had higher protein interaction numbers. Among them, *CDK1* has important functions in the coupling of cell proliferation and protein synthesis. CHEK1, as an important regulator, affects cell cycle arrest in the G2 phase, while DNA damage regulates cell cycle and apoptosis by acting on *CDK1* and Chek1 [44,45,46]. *CDC6* and *CDC45* have the important function in the immune process of adjusting their own expression levels [47]. The coordinated upregulation and network prominence of these core cell cycle regulators suggest their involvement in modulating critical cellular processes during prolonged salinity stress in *S. esculenta*. This response represents an adaptive mechanism to manage cell proliferation, DNA replication, and genome stability under sustained osmotic challenge. The significant involvement of cell cycle regulators such as *CDK1*, *CHEK1*, and *CDC* genes under prolonged stress illustrates a critical layer of immune-cell proliferation and genomic integrity maintenance. As immune responses often require clonal expansion and rapid cell turnover, these genes support immunocompetence by ensuring orderly progression of the cell cycle under stress. Their adaptive expression underscores a mechanism by which *S. esculenta* preserves immune functionality and cellular viability during sustained environmental challenge. Their upregulation suggests a stress-induced reorganization of cell cycle activities that may support immune cell turnover and tissue repair under long-term different salinity stress.

### 4.6. Review of Key Gene Sets and Genes Under Different Salinity Stress

In our research, we found that the chemokine signaling pathway gene set was activated in all four experimental groups and may have more important biological significance under different time and salinity stress. Under the four stresses, *NFKBIA*, as the core gene of the gene set, may be used as a candidate factor, potentially mitigating oxidative stress and modulating immune function. Complementary physiological data demonstrated that both high- and low-salinity stress significantly enhanced immune competence and antioxidant capacity in *S. esculenta*, consistent with the functional predictions derived from the core genes and enrichment analyses. The identified hub genes, characterized by their central positions, are strongly implicated in mediating *S. esculenta*’s adaptation to environmental stressors. In the earlier research, the function of key genes such as *NFKBIA* has not been widely studied in *S. esculenta*.

## 5. Conclusions

**Figure 9:**
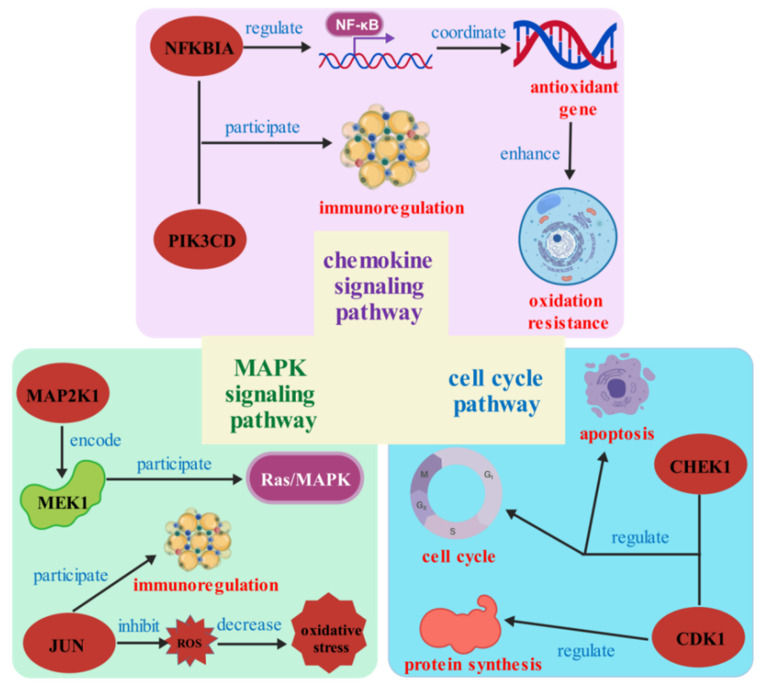
Regulation mechanism of core genes with more protein interactions in the face of salinity stress.

In our research, we explored the molecular response of *S. esculenta* under different time and salinity stresses. The results of GO and KEGG enrichment analysis identified a large number of GO terms and signaling pathways related to immunity and signal transduction. Combined with physiological indicators, the response mechanism of *S. esculenta* was further supplemented and verified. Complementary physiological assays validated the activation of stress-responsive systems, aligning with molecular profiling data. More importantly, the chemokine signaling pathway gene set was significantly enriched in SAL40_4h, SAL20_24h, and SAL40_24h. The chemokine signaling pathway exhibited consistent enrichment across SAL40_4h, SAL20_24h, and SAL40_24h, suggesting its fundamental role in osmotic adaptation. We speculate that the gene set may have important biological significance when the *S. esculenta* faces salinity stress at different times. We constructed a mechanism map using the core genes with a high number of protein interactions in the above gene sets to express our speculation on the regulatory mechanism of these genes in the face of salinity stress (Figure 9). *NFKBIA*s upregulation positions it as a key candidate for mitigating oxidative damage during salinity adaptation. *NFKBIA* is the core gene in the gene set of the chemokine signaling pathway under different time and salinity stresses, indicating that this gene may protect the body from oxidative damage when *S. esculenta* faces salinity stress, but its specific mechanism of action needs further study. By linking transcriptomics with physiological verification, we propose a scientific hypothesis to improve the efficiency of aquaculture productivity and breeding of excellent varieties in the context of global environmental changes. Our results provide support for the study of the stress response mechanism of *S. esculenta* and contribute to the selection of excellent varieties of *S. esculenta* and further large-scale breeding.

## Figures and Tables

**Table 1:** List of primers used for quantitative RT-PCR validation.

Gene Name	Forward Primer (5′-3′)	TM (°C)	Reverse Primer (5′-3′)	TM (°C)	Amplicon Length (bp)
*CDC16*	TAGCAAAGCGACCACATTAG	60	CTGGGAGGCAGTGAAATAAG	60	118
*CDC25B*	CTGCCAATAAAGACTGCTATCC	61	CTCACACATTTCCGTTCCAC	61	101
*CDC42*	GTTGTGGTTGGTGATGGT	60	GCCCTGCAGTATCAAAGAG	60	163
*CDC45*	CCCGATGATGATGTCTGTTT	60	CTGGGATGTCTCTCATTTCTTC	60	121
*CDC6*	CAGGTGGCAGCCTTTATATT	60	CTCACTCCCATGCAGTTAAG	60	130
*CDK1*	GCCAGGAGTAACCTCATTAC	60	AAGTCCTGCGTCATCTAATC	59	100
*CHEK1*	CTTGGAGAAGGTGCTTATGG	60	CCCTGACAGAAACCTCTTTATC	60	106
*DUSP1*	GCAGACGTAGCGTCATTT	60	GGGAGTGATGATGTCCTAAAC	60	172
*DUSP10*	CGGTGCGAATATCCTTATCC	60	CGCTTCTGTTTCACGTATCT	60	126
*DUSP7*	CCCTTAACGTCCTTGGTATT	59	GCTGGATGGATTCTCGTTTA	60	125
*ESPL1*	AGCACCACCAGTAGAGATAA	60	CTCTTGGCAGTCCTTTAGTG	60	117
*FLNA*	TGACAGAAGTGCTGCTAAAC	60	GGTTCGAGAGGCATGTAATC	60	123
*GSK3B*	TGTGCCTGAGACTGTGTA	60	CTCCTTGGGAATGGATGTATG	60	128
*JUN*	ATGTGACCGAAGAACAAGAG	60	GTGGAGGCGATGTAGAATTT	60	102
*MAD2L1*	GGCTTATAGAGATGGTGTTGG	60	CCAGTGTCATCTTCCACATC	60	103
*MAP2K1*	CCATGACTGGGAAACCTTTA	60	AGGGTGGAGGCTCATTTA	60	129
*MAPKAPK2*	GCCCAAATCACGAAGAGAA	60	CAACAAGGAAGGCACACT	60	112
*MRAS*	CTTGCCGCTCACCTTAAT	60	GTGGCTGCTCTCGAATAAT	60	109
*MYC*	CACCTGGAACGAGAAAGAAG	60	TCGTAGCCTCATCCACAA	60	121
*NFKB1*	GCCTGCATAAAGCTGTTAGA	60	AGAGCAGTCTGGGACTTT	60	127
*NFKBIA*	CTGCCCTCCAGAAACATTAC	60	CCCATGTTCAGCAGCATAA	60	116
*PIK3CA*	GCAGGTGATCAGGATTATGG	60	GCAGCAGGAACAACTTCA	60	120
*PIK3CD*	GGTGTTGTACGTGATAGAGTG	60	CGGCTGCTGGAACAAATA	60	131
*PIK3R1*	GGCGAGTTATCAAGAGAAGAG	60	TCTAAGGGTCAGAGTGTAGTC	60	108
*PTK2*	GAAGTCACGAGAAAGCTACTG	60	CCGAAGTTCATACCTCCATTC	60	106
*RAC2*	AGGCAATTGGTGGGTTAG	59	CGCAGAACACTCCATGTAT	60	107
*ROCK1*	TTGTATGCGGATGGATGTG	60	CATCACCACCTTGGGATTT	60	101
*SOS1*	CAGGAAGCAAGTGGAGAAAG	61	GAACTGTAAGTGGTGAGGAATG	61	125
*TFDP1*	GACACATCCTACCTCCTTTATG	60	CAACATTTGGGCTTTGATCC	60	133
*VAV1*	CAGTGATGACGAGGACATCTA	61	CTTCCTCTTCGTACACAACATC	61	101

**Table 2:** RNA-Seq results.

Sample	Raw Reads	Raw Bases	Clean Reads	Clean Bases	Q20 (%)	Q30 (%)	Total Mapping
C_0h_1	461,138,06	6.92G	449,821,04	6.75G	97.07	92.32	39456868 (87.72%)
C_0h_2	505,451,56	7.58G	493,877,00	7.41G	97.44	93.02	44052010 (89.20%)
C_0h_3	475,155,46	7.13G	463,599,16	6.95G	97.27	92.68	41025164 (88.49%)
C_4h_1	461,799,90	6.93G	451,241,90	6.77G	96.77	91.61	39813899 (88.23%)
C_4h_2	464,091,28	6.96G	449,101,60	6.74G	97.27	92.62	40048934 (89.18%)
C_4h_3	443,214,76	6.65G	430,526,24	6.46G	96.27	90.81	37441672 (86.97%)
C_24h_1	447,868,92	6.72G	431,101,54	6.47G	95.85	90.05	37201612 (86.29%)
C_24h_2	449,431,88	6.74G	433,935,64	6.51G	95.83	90.02	37413049 (86.22%)
C_24h_3	457,950,96	6.87G	448,865,64	6.73G	97.42	92.92	39924459 (88.95%)
SAL20_4h_1	460,619,26	6.91G	448,902,60	6.73G	97.06	92.29	39336186 (87.63%)
SAL20_4h_2	459,147,26	6.89G	445,807,56	6.69G	97.22	92.65	39383060 (88.34%)
SAL20_4h_3	501,220,24	7.52G	486,853,20	7.30G	95.69	89.74	42000224 (86.27%)
SAL20_24h_1	426,766,10	6.40G	415,836,98	6.24G	97.26	92.68	36631715 (88.09%)
SAL20_24h_2	476,870,56	7.15G	463,630,26	6.95G	97.36	92.84	40997032 (88.43%)
SAL20_24h_3	464,417,70	6.97G	451,153,78	6.77G	97.41	92.91	39895803 (88.43%)
SAL40_4h_1	466,644,44	7.00G	448,498,10	6.73G	97.45	93.00	39852403 (88.86%)
SAL40_4h_2	412,505,16	6.19G	401,588,60	6.02G	95.80	89.92	34776949 (86.60%)
SAL40_4h_3	455,434,02	6.83G	440,828,56	6.61G	97.33	92.81	39194280 (88.91%)
SAL40_24h_1	459,581,10	6.89G	447,316,52	6.71G	97.52	93.10	39903972 (89.21%)
SAL40_24h_2	477,382,44	7.16G	465,891,80	6.99G	97.49	93.38	41137815 (88.30%)
SAL40_24h_3	476,587,96	7.15G	465,246,48	6.98G	97.21	92.51	41317022 (88.81%)

## Data Availability

Data will be made available on request.

## Supplementary Materials

The following supporting information can be downloaded at: https://www.mdpi.com/article/10.3390/biology14101338/s1, Table S1: Level-3 KEGG signaling pathways of DEGs.

## Abbreviations

The following abbreviations are used in this manuscript:

*S. esculenta*	*Sepia esculenta*
GO	Gene Ontology
KEGG	Kyoto Encyclopedia of Genes and Genomes
DEGs	Differentially expressed genes
qRT-PCR	quantitative Reverse Transcription Polymerase Chain Reaction
PPI	Protein-Protein Interaction
GSEA	Gene Set Enrichment Analysis
