# Supplementary material for: Salinity Stress Mechanisms in Sepia esculenta Larvae Revealed by Integrated Biochemical and Transcriptome Analyses
Source: Biology (Basel). 2025 Sep 30;14(10):1338. doi: 10.3390/biology14101338 (PMC12561133; doi:10.3390/biology14101338)
Supplement: Supplementary file 1 [file biology-14-01338-s001.zip › biology-3879035-Supplementary.pdf]

Table S1 Level-3 KEGG signaling pathways of DEGs.

A: KEGG signaling pathways were enriched with DEGs under SAL20_4h stress.

Pathway	Number of DEGs
Apoptosis	3
Cholesterol metabolism	3
Relaxin signaling pathway	3
HIF-1 signaling pathway	3
Protein digestion and absorption	3
Axon guidance	3
Neuroactive ligand-receptor interaction	4
Neutrophil extracellular trap formation	3
2-Oxocarboxylic acid metabolism	2
NOD-like receptor signaling pathway	2
Biosynthesis of amino acids	2
Ascorbate and aldarate metabolism	2
Arginine biosynthesis	2
cAMP signaling pathway	4
PI3K-Akt signaling pathway	3
MAPK signaling pathway	3

B: KEGG signaling pathways were enriched with DEGs under SAL20_24h stress.

Pathway	Number of DEGs
DNA replication	6
Cell cycle	5
MAPK signaling pathway	6
Metabolic pathways	19
Oxytocin signaling pathway	3
Axon guidance	3
2-Oxocarboxylic acid metabolism	2
Biosynthesis of amino acids	2
Glutamatergic synapse	2
Carbon metabolism	2
Glyoxylate and dicarboxylate metabolism	2
Neutrophil extracellular trap formation	3
Mineral absorption	2
Ovarian steroidogenesis	2
Insulin secretion	2
Glutathione metabolism	2

C: KEGG signaling pathways were enriched with DEGs under SAL40_4h stress.

Pathway	Number of DEGs
Phospholipase D signaling pathway	3
Ras signaling pathway	2
Glutamatergic synapse	3
Notch signaling pathway	2
Base excision repair	2
Fanconi anemia pathway	2
ECM-receptor interaction	2
Efferocytosis	3
Inositol phosphate metabolism	2
Osteoclast differentiation	2
Protein digestion and absorption	2
Apoptosis	2
Cellular senescence	2
cAMP signaling pathway	2
PI3K-Akt signaling pathway	2
MAPK signaling pathway	2

D: KEGG signaling pathways were enriched with DEGs under SAL40_24h stress.

Pathway	Number of DEGs
DNA replication	5
Sphingolipid metabolism	4
Oocyte meiosis	3
AMPK signaling pathway	5
Motor proteins	5
Apoptosis	4
Mismatch repair	3
2-Oxocarboxylic acid metabolism	2
Metabolic pathways	18
Carbon metabolism	2
Cytoskeleton in muscle cells	4
PI3K-Akt signaling pathway	5
Notch signaling pathway	2
p53 signaling pathway	2
Cell cycle	4
MAPK signaling pathway	4
